# High specificity of line-immunoassay based algorithms for recent HIV-1 infection independent of viral subtype and stage of disease

**DOI:** 10.1186/1471-2334-11-254

**Published:** 2011-09-26

**Authors:** Jörg Schüpbach, Leslie R Bisset, Stephan Regenass, Philippe Bürgisser, Meri Gorgievski, Ingrid Steffen, Corinne Andreutti, Gladys Martinetti, Cyril Shah, Sabine Yerly, Thomas Klimkait, Martin Gebhardt, Franziska Schöni-Affolter, Martin Rickenbach

**Affiliations:** 1 University of Zurich, Institute of Medical Virology, Swiss National Center for Retroviruses, Zurich, Switzerland; 2 Zurich University Hospital, Clinic for Immunology, Zurich, Switzerland; 3 University Hospital Lausanne and University of Lausanne, Division of Immunology, Lausanne, Switzerland; 4 University of Berne, Institute of Infectious Diseases, Berne, Switzerland; 5 University of Basel, Institute for Medical Microbiology, Basel, Switzerland; 6 Clinique de la Source, Laboratory, Lausanne, Switzerland; 7 Istituto Cantonale di Microbiologia, Bellinzona, Switzerland; 8 University Hospital of Geneva and University of Geneva Medical School, Laboratory of Virology, Geneva, Switzerland; 9 Swiss Federal Office of Public Health, Berne, Switzerland; 10 Swiss HIV Cohort Study (SHCS) Data Center, University Hospital Lausanne, Lausanne, Switzerland

## Abstract

**Background:**

Serologic testing algorithms for recent HIV seroconversion (STARHS) provide important information for HIV surveillance. We have shown that a patient's antibody reaction in a confirmatory line immunoassay (INNO-LIA^TM^ HIV I/II Score, Innogenetics) provides information on the duration of infection. Here, we sought to further investigate the diagnostic specificity of various Inno-Lia algorithms and to identify factors affecting it.

**Methods:**

Plasma samples of 714 selected patients of the Swiss HIV Cohort Study infected for longer than 12 months and representing all viral clades and stages of chronic HIV-1 infection were tested blindly by Inno-Lia and classified as either incident (up to 12 m) or older infection by 24 different algorithms. Of the total, 524 patients received HAART, 308 had HIV-1 RNA below 50 copies/mL, and 620 were infected by a HIV-1 non-B clade. Using logistic regression analysis we evaluated factors that might affect the specificity of these algorithms.

**Results:**

HIV-1 RNA <50 copies/mL was associated with significantly lower reactivity to all five HIV-1 antigens of the Inno-Lia and impaired specificity of most algorithms. Among 412 patients either untreated or with HIV-1 RNA ≥50 copies/mL despite HAART, the median specificity of the algorithms was 96.5% (range 92.0-100%). The only factor that significantly promoted false-incident results in this group was age, with false-incident results increasing by a few percent per additional year. HIV-1 clade, HIV-1 RNA, CD4 percentage, sex, disease stage, and testing modalities exhibited no significance. Results were similar among 190 untreated patients.

**Conclusions:**

The specificity of most Inno-Lia algorithms was high and not affected by HIV-1 variability, advanced disease and other factors promoting false-recent results in other STARHS. Specificity should be good in any group of untreated HIV-1 patients.

## Background

Information on HIV incidence is necessary for monitoring the dynamics of the HIV epidemic in affected countries and assessing the effectiveness of preventive measures targeted at major risk populations. Consequently, serologic testing algorithms for recent HIV seroconversion (STARHS) have been developed. These tests make use of the fact that both the concentration and affinity of HIV antibodies during the first few months of HIV infection are lower than at later stages [1-4]. STARHS require a special assay of reduced sensitivity, hence they are also called 'detuned' assays. The reduced sensitivity renders these tests unsuitable for diagnosis of HIV infection and restricts their use to epidemiologic studies. For a systematic epidemiologic monitoring it would be advantageous if information on the proportion of recent infections could be gained prospectively and systematically from the tests used anyway to diagnose HIV infection.

We have shown that a patient's antibody reaction in a commercial line immunoassay, the Inno-Lia^TM^ HIV I/II Score (Inno-Lia), provides information on the duration of infection similar to that of a commercial enzyme immunoassay (EIA) for STARHS, the so-called BED-EIA [5,6]. The Inno-LIA is a kind of second-generation Western blot and measures antibodies to different HIV antigens in a semi-quantitative way. The pattern and intensity of HIV-specific antibodies both evolve during the first weeks to months after infection. It is thus possible to define algorithms which, with a certain diagnostic sensitivity and specificity, recognize early and late antibody patterns. Based on the number of cases ruled recent by the Inno-Lia and the known values for sensitivity and specificity it is then possible to calculate the proportion of infections of up to 12 months duration in a group by using a simple formula [5]. As the Inno-Lia is a confirmatory HIV test, it is convenient to prospectively test all newly diagnosed patients and to notify the results to the respective health authority, which will calculate periodically the proportion of recent infections among the different transmission risk groups.

The diagnostic sensitivity and specificity of each algorithm are crucial for this method. If they are not correct, estimates of recent infections will not be accurate. We estimated these parameters for a total of 12 algorithms in a baseline study of newly diagnosed patients with HIV-1 infection of either less or more than 12 months duration, as judged by the treating physicians of these patients. The estimates for sensitivity resulting from this study varied between 20 and 50%, while specificity was between 92 and 100%. The algorithm, which distinguished best between incident and older infection, had a sensitivity of 50.3% and a specificity of 95.0% [5]. As the study was prospective, it was difficult to know whether the treating physician's judgment on the duration of each infection was correct. Follow-up information on the patients on the course of HIV-1 RNA and CD4+ T cell concentrations over time, which is sometimes necessary for differentiating between severe primary and advanced HIV infection, was not available at the time of diagnosis, and the reliability of the staging information in that first study is therefore somewhat arguable. For example, some of the patients classified as CDC stage B or C by the diagnosing physician, but ruled recent by the Inno-Lia algorithms, may actually have suffered from severe acute HIV infection [7-9]. Another well-known cause for a false classification is infection by non-B subtypes of HIV-1 [10,11]. Patients infected with non-B viruses may produce antibodies of reduced avidity to the subtype B antigens frequently employed in serologic tests, thus leading to false classification as recent. Similarly, the waning antibody titers to some HIV proteins in advanced immunodeficiency may lead to false classification as recent infection [12-17].

Reliable information on the diagnostic performance of our method is thus still lacking, and the true diagnostic sensitivity and specificity of the Inno-Lia algorithms for recent infection still have to be established. Towards this goal, we have conducted two studies. One study, to be published elsewhere, will determine the diagnostic sensitivity in a cohort of patients diagnosed at the time of primary HIV infection. That study will also investigate the overall diagnostic performance of the algorithms and present a validation of the method in consecutive annual cohorts of HIV notifications.

In contrast, the goal of the present study was to further assess the specificity of the algorithms in HIV-1 patients known to have been infected for longer than 12 months and to identify factors that might influence the outcome of the algorithms. Of particular interest was the question whether there was an impact by the HIV-1 subtype or an advanced stage of disease.

## Methods

### Ethics statement

This study was conducted as a nested project in the framework of the Swiss HIV Cohort Study (SHCS; see http://www.shcs.ch ) [18]. The ethical committees of all participating institutions, i.e., the HIV outpatient clinics and laboratories of seven Swiss hospitals (the university hospitals of Basel, Berne, Geneva, Lausanne, Zurich, and the cantonal hospitals of Lugano and St Gallen), have approved the general study protocol, and all participating patients have given their written informed consent to the goals of the SHCS and its research projects, including this one.

### Patients and samples

The study investigated a single plasma or serum specimen from a total of 714 patients of the SHCS. The patients and their specimens were selected for the study in spring 2008. All patients had been infected with HIV-1 for at least 12 months, as demonstrated by either a documented first positive HIV test or registration into the SHCS at least 12 months prior to sample date. The patients originated from 7 different SHCS treatment centers and represented all clinical stages and CD4+ strata. The HIV-1 subtype of all patients was known based on the recorded results of genetic resistance testing in the reverse transcriptase and protease regions of the *pol *gene. Patients were selected with the aim that all viral subtypes and circulating recombinant forms (CRF) were represented with 30 samples both in CDC stages A and B and with 40 samples in stage C. If there were more patients of a given subtype per stage, the patients required were selected randomly. If there were fewer, all available were selected. Of all but 12 patients, a plasma aliquot stored at -70°C was used for testing. For 12 patients a frozen serum sample stored at -20°C was used instead.

### Serological differentiation of recent and older HIV-1 infection

All samples were number-coded and tested retrospectively, batch-wise by the Inno-Lia^TM^ HIV I/II Score assay (Innogenetics, Ghent, Belgium). Testing was conducted in 7 different accredited laboratories including 6 HIV regional confirmatory laboratories commissioned by the Swiss Federal Office of Public Health (SFOPH) and the Swiss National Center for Retroviruses (SNCR), which serves as the national HIV reference laboratory and is also commissioned by the SFOPH. All 7 labs are accredited according to the international standard ISO/IEC 17025 by the governmental Swiss Accreditation Service SAS (see http://www.seco.admin.ch/sas/index.html?lang=en). All had participated already in the first study of Inno-Lia based recent infection assessment and were experienced with the test [5].

The Inno-Lia is a Western blot-like line immunoassay that measures antibodies against recombinant proteins or synthetic peptides of HIV-1 group M, HIV-1 group O, or HIV-2, which are coated as 7 discrete lines on a nylon strip with plastic backing. As each test strip also contains three quantitative internal standards, a semi-quantitative ranking of the different antibody reactions is possible [19,20].

All assays were performed between Oct 2008 and Jan 2009 and involved 4 different lots of test kits. The manufacturer's 16-h sample incubation protocol was used for all tests. In 3 labs, on a total of 498 samples, testing was conducted on CE-marked Auto-Lia 48 or Autoblot 3000 test automats (both from Innogenetics). In 4 labs, on a total of 216 samples, testing was performed manually. Antibody reaction to each of the 7 HIV antigen bands present on the test strips (sgp120 [including group O peptides], gp41, p31, p24 and p17 of HIV-1, and sgp105 and gp36 of HIV-2) was assessed either visually (in three of the four labs that used manual testing on a total of 123 samples) or by the automated scanner-based LiRAS system (Innogenetics) (in 4 labs; 591 samples). Based on the three internal standards, which define reaction levels of 0.5 (+/-), 1 and 3 for each test strip, the antibody reaction to each HIV antigen was classified into one of six possible intensity scores (0, 0.5, 1, 2, 3, or 4).

### Inno-Lia algorithms

Twenty-four algorithms (Algs) for recent HIV-1 infection were developed empirically by investigating which Inno-Lia antibody patterns were found at maximal frequency in a group of patients with less than 12 months of infection (= recent or incident infections) and at minimal frequency in a group of patients with ≥12 months duration of infection (= older infections). Twelve of the algorithms, Alg02 to Alg13, are as published [5]. The other 12 were developed more recently based on the same dataset [5]. All 24 algorithms were applied to the collected Inno-Lia data. Thus, each Inno-Lia result was classified by 24 algorithms as representing either a recent or older HIV-1 infection.

### Data evaluation and statistics

The results of Inno-Lia testing and the clinical data of the SHCS were linked only after all testing was completed. Differences between means were analyzed by the nonparametric Mann-Whitney U test, differences in frequency by contingency tables and Fisher's exact test, and correlations by nonparametric Spearman's rank correlation. Predictors of result of Inno-Lia algorithms (incident or older infection) were evaluated by univariate and multivariate logistic regression analysis. Independents analyzed included person-related parameters (sex, age, time since registration into the SHCS), disease-related factors (CDC stage, CD4+ T-cell count and percentage, treatment status, duration of HAART, HIV-1 RNA concentration as by commercial RT-PCR assays from Roche), and testing modalities (type of specimen, storage duration, lot number of test kit, modes of testing and result evaluation, laboratory which stored the samples and performed the testing). All statistical analyses, as well as the classification of the Inno-Lia results by the 24 recent infection algorithms, were performed in the StatView 5.0 program for Macintosh (SAS Institute, Cary, North Carolina, U.S.A.).

## Results

A total of 714 stored plasma or serum samples from patients who participated in the SHCS and had been infected by HIV-1 for at least 12 months were tested by the Inno-Lia HIV I/II score assay, as described under Methods. The main epidemiological, virological and immunological characteristics of the patients are summarized in Table 1. Owing to the selection for non-B clade infections, which in our country are more frequent in women than in men, the two sexes were represented at about equal numbers. Roughly half of the patients were classified as CDC stage A, 22% were in stage B and 28% in stage C. Almost three quarters of the patients had received HAART for a median duration of 13.5 months, resulting in 308 patients who presented with a HIV-1 RNA concentration below 50 copies/mL. HIV-1 RNA among the 406 patients with HIV-1 RNA ≥50 copies/mL amounted to a median of 10^3.94 ^copies/mL. The majority of the patients (86.8%) were infected by non-B clades comprising a total of 15 different clades in addition to subtype B.

**Table 1:** Patient characteristics

Patients (n, %)	714	100
Male (n, %)	345	48.3
Female (n, %)	369	51.7
Age, years (median, IQR)	35	30 - 42
Clinical stage		
CDC A (n, %)	354	49.6
CDC B (n, %)	158	22.1
CDC C (n, %)	202	28.3
CD4+ T cell count, cells/μL (median, IQR)	350	220 - 533
CD4+ T cells, percent (median, IQR)	21.0	15.0 - 29.0
CD8+ T cell count, cells/μL (median, IQR)	808	565 - 1133
CD8+ T cells, percent (median, IQR)	51.0	41.0 - 59.0
Patients with HIV-1 RNA <50 copies/mL (n, %)	308	43.1
Patients with HIV-1 RNA ≥50 copies/mL (n, %)	406	56.9
HIV-1 RNA among these (log [copies/mL], IQR)	3.94	2.97-4.68
HIV-1 clade (n, %)		
B	94	13.2
Non-B (15 different clades)	620	86.8
Treatment status		
HAART-naive (n, %)	190	26.6
Receiving HAART (n, %)	524	73.4
Months on HAART if receiving HAART (median, IQR)	13.5	11.1 - 17.8

### Influence of HAART

**Figure 1:**
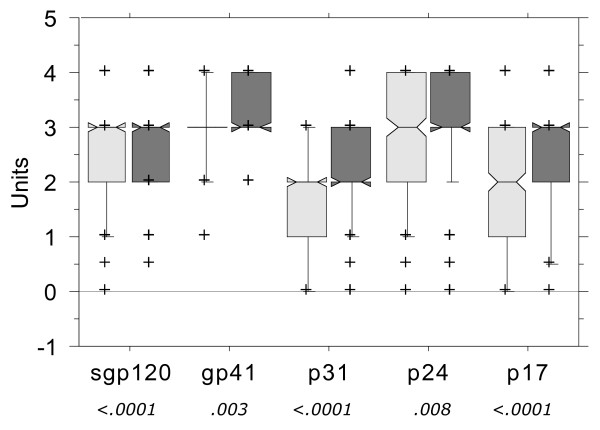
**Effect of concentration of HIV-1 RNA on intensity of Inno-Lia bands**. The box-plots indicate the median (the "waist" of the boxes) and the quartiles (upper and lower boundary of the boxes); outliers above the 90^th ^or, respectively, below the 10^th ^percentile (horizontal lines outside of the boxes) are plotted individually as crosses. Numbers at the bottom indicate the p-values of the Mann-Whitney U test for differences between patients with HIV-1 RNA <50 copies/mL (lightly shaded; 308 patients) and ≥ 50 copies/mL (darkly shaded; 406 patients).

The group of patients receiving HAART at the time of testing had significantly lower concentrations of HIV-1 RNA than those untreated (10^1.59 ^copies/mL compared to 10^4.25 ^copies/mL; p < 0.0001, Mann-Whitney U test). They also had significantly less intense reactions in the Inno-Lia with respect to viral proteins sgp120 (p = 0.029), p31 (p < 0.0001), p24 (p = 0.0003) and p17 (p = 0.0013). In contrast, the intensity of antibodies to gp41 was similar in both treated and untreated patients (p = 0.17), and the minimal band intensity was 1.0 independent of the treatment status. There was also a strong association between viral load and band intensity (Figure 1). The 308 patients (including 6 who were treatment-naïve) with HIV-1 RNA <50 copies/mL had on average significantly lower intensity of all bands than the 406 patients with ≥50 copies/mL, particularly with respect to sgp120, p31 and p17 (p < 0.0001 for all three) and somewhat less with respect to gp41 (p = 0.003) and p24 (p = 0.008). The result of the Inno-Lia algorithms, recent or older infection, was affected likewise by treatment status and viral load (not shown). Multivariate logistic regression analysis combining HIV-1 RNA, treatment status and duration of HAART as independents showed, however, that the treatment status and the duration of HAART had no significant effect on the result of any algorithm. In contrast, the viral load level - more or less than 50 copies/mL - was a significant determinant for outcome of all algorithms except Alg03, Alg03.1, Alg05 and Alg06 (data not shown).

Based on these first findings we determined the diagnostic specificity of the 24 Inno-Lia algorithms among the 190 treatment-naïve patients (including 6 patients with HIV-1 RNA <50 copies/mL) and the 222 patients with a viral load ≥50 copies/mL despite receiving HAART. Algorithm specificity among these 412 patients extended from 92.0% to 100%, with a median of 96.5% (Table 2). Perfect specificity (100%) was obtained with the single-band algorithms Alg03 and Alg03.1; Alg06 was least specific (92.0%). Specificity of the algorithms among the 190 HAART-naïve patients alone was similar (median 95.5%, range 93.2 -- 100%).

**Table 2:** Specificity of 24 Inno-Lia algorithms among 412 patients either HAART-naïve or exhibiting HIV-1 RNA ≥50 copies/mL despite HAART

Alg #	Definition	N recent	Specificity %
* Single-band algorithms*			

2	if sgp120 ≤ 1 then RECENT else older	16	96.1

3	if gp41 ≤.5 then RECENT else older	0	100

3.1	if gp41 ≤ 1 then RECENT else older	0	100

3.2	if gp41 ≤ 2 then RECENT else older	6	98.5

4	if p31 = 0 then RECENT else older	25	93.9

4.1	if p31 ≤ 0.5 then RECENT else older	28	93.2

5	if p24 ≤ 0 then RECENT else older	9	97.8

6	if p17 = 0 then RECENT else older	33	92.0

* Combined algorithms*			

7	if sgp120 + gp41 + p31 ≤ 4 then RECENT else older	7	98.3

8	if gp41 ≤ 0.5 OR (sgp120 + gp41 + p31 ≤ 4) OR (sgp120 + gp41 + p31 + p24 + p17≤ 6.5) then RECENT else older	13	96.8

8.1	if gp41 ≤ 0.5 OR (sgp120 + gp41 + p31 ≤ 4) OR ((sgp120 + gp41 + p31 + p24 + p17≤ 6.5) AND p31≤1) then RECENT else older	13	96.8

9	if sgp120 + gp41 ≤ 4 AND p31 = 0 then RECENT else older	7	98.3

10	if p31 = 0 AND p24 ≥ 2 then RECENT else older	16	96.1

11	if (sgp120 + gp41 ≤ 2.5) OR (sgp120 + gp41 + p31 + p24 + p17 ≤ 6.5) OR (p31 = 0 AND p24 ≥ 2) then RECENT else older	23	94.4

11.1	if (sgp120 + gp41 ≤ 2.5) OR ((sgp120 + gp41 + p31 + p24 + p17 ≤ 6.5) AND p31≤1) OR (p31 = 0 AND p24 ≥ 2) then RECENT else older	23	94.4

12	if (p24 ≥ 2 AND p31 = 0) OR (gp41 ≤.5) OR (sgp120 + gp41 + p31 ≤ 4 OR sgp120 + gp41 + p31 + p24 + p17 ≤ 6.5) then RECENT else older	23	94.4

12.1	if (p24 ≥ 2 AND p31 = 0) OR (gp41 ≤.5) OR (sgp120 + gp41 + p31 ≤ 4) OR (p31 ≤ 1 AND (sgp120 + gp41 + p31 + p24 + p17 ≤ 6.5)) then RECENT else older	23	94.4

13	if (sgp120 + gp41 ≤ 4 AND p31 = 0) OR (p31 = 0 AND p24 ≥ 2) then RECENT else older	17	95.9

13.1	if gp41 ≤ 2 OR (p31 = 0 AND p24 ≥ 2) then recent else older	20	95.1

14	if (sgp120 + gp41 + p31 + p24 + p17 ≤ 6.5 AND p31 ≤ 1) then RECENT else older	8	98.1

15	if (sgp120 ≤ 1 AND p31 ≤ 1) OR (gp41 ≤ 2 AND p31 ≤ 1) OR (p17 ≥ 2 AND p31 = 0) OR (p31 = 0 AND p24 ≥ 2) then RECENT else older	18	95.6

16	if (sgp120 ≤ 1 AND (p31 + p24 + p17 ≤ 2.5)) OR (gp41 ≤ 1 ) OR (p31 ≤ 0.5 AND (sgp120 + gp41+ p24 + p17 ≥ 15)) OR (p24 = 0 AND gp41 ≤ 2 ) OR (p24 ≥ 3 AND p31 = 0 ) then RECENT else older	12	97.1

17	if (sgp120 * gp41) ≤ 2 then RECENT else older	4	99.0

18	if (sgp120 * gp41 ≤ 1) OR (p24 + p31 = 0) then RECENT else older	5	98.8

### Investigation of factors that affect algorithm specificity

**Figure 2:**
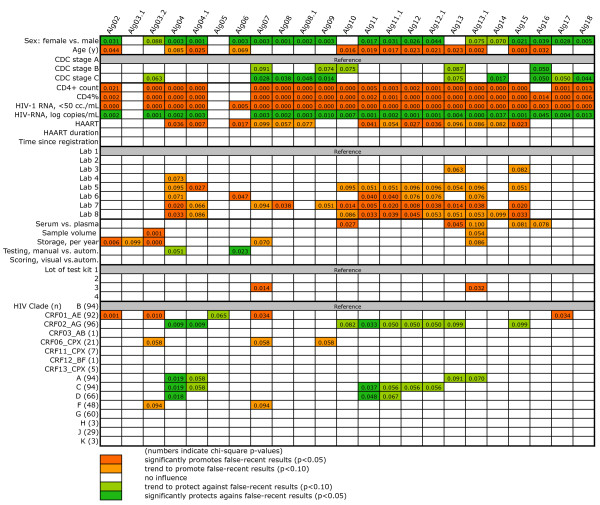
**Univariate logistic regression analysis of factors that promote or impair algorithm specificity in all 714 patients**. The meaning of the colors is explained at the bottom of the figure. Numbers indicate the chi-square p-value of the respective variable analyzed. HIV-1 RNA was used as a dichotomized parameter (<50 or ≥50 copies/mL).

**Figure 3:**
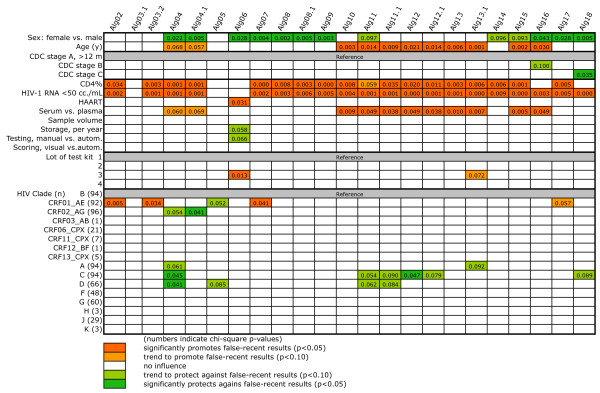
**Multivariate logistic regression analysis of factors that affect algorithm specificity in all 714 patients**. The meaning of the colors is explained at the bottom of the figure. Numbers indicate the chi-square p-value of the respective variable analyzed. Odds ratios of variables of particular interest and their 95% confidence intervals are shown in the text. HIV-1 RNA was used as a dichotomized parameter (<50 or ≥50 copies/mL).

Using logistic regression analysis, we sought to identify the factors that affected the result of the various algorithms in the total of the 714 patients. Alg03 could not be analyzed, as it was 100% specific. Results for the remaining 23 algorithms are summarized in Figures 2 and 3. There were predictors that promoted false-recent results and others which protected against these. Most of the effects were not distributed randomly, but were associated with distinct groups of algorithms.

In the univariate analysis (Figure 2), the strongest and most consistent predictors of algorithm result included the HIV-1 RNA level, the CD4+ T cell percentage (CD4%) or count, sex, HAART status, age, and CDC stage. HIV-1 RNA <50 copies/mL, CD4% or count, age, and receiving HAART promoted a recent infection result. Other promoting factors included, in decreasing order, testing in certain laboratories compared to the one taken as reference, a long duration of sample storage, or being infected with the circulating recombinant form (CRF) CRF01_AE. Conversely, HIV-1 RNA concentration (in log(copies/mL), female sex or, for some algorithms, being in CDC stages B or C compared to A were factors that protected against a recent infection result. Other protective factors for some algorithms included being infected with CRF02_AG or subtypes A, C or D, or manual Inno-Lia testing. There were also some sporadic associations with the type or volume of the stored specimen or lot number of test kit. No associations were seen for duration of HAART, time since registration into the SHCS and mode of result scoring (visual versus automated).

The multivariate analysis of factors that affected algorithm specificity (Figure 3) was performed with all parameters that had shown at least one significant association in the univariate analysis. There was strong co-linearity between *CD4 count *and *CD4%*, the two parameters for HIV-1 RNA, as well as *testing laboratory *and *mode of testing*. We therefore excluded *CD4 count *and *testing laboratory *from the analysis. Regarding HIV-1 RNA, we excluded log(copies/mL) in favor of the statistically stronger level.

The multivariate analysis confirmed the importance of HIV-1 RNA, CD4%, sex and age. Specifically, for the 20 algorithms for which an effect of HIV-1 RNA was demonstrated, <50 copies/mL was associated with a roughly fivefold increase in false-recent results compared to a concentration ≥50 copies/mL (odds ratio [OR]; mean, 4.85; range, 3.1 - 45.5). For the 18 algorithms affected by CD4%, there was a mean 1.046fold (range 1.025 - 1.083) increase in false-recent results for each additional CD4%, i.e. by 4.6%. Women had a mean 2.4fold lower risk than men for the 13 affected algorithms (mean OR, 0.412; range, 0.203 - 0.620). For age, there was a 3.2% increase of false-recent results per additional year with respect to the 11 marked algorithms (mean OR, 1.032; range 1.021 - 1.043).

Furthermore, for those algorithms in which age promoted a false-recent result, the testing of serum stored at -20°C instead of plasma stored at -70°C appeared to be a further promoting factor. There were only 12 serum samples, however, thus relativizing this finding. Advanced clinical stage lost the protective effect seen in univariate analysis in all algorithms but one. Sample size, duration of sample storage, and modes of testing and result evaluation retained no significance. Test kit lot #3 was again associated with a lower specificity when using Alg07 or, as a trend, Alg13.1. Close inspection of the data showed, however, that the great majority of the samples, namely 671 (86.4%), had been tested with lot #1. Only 32 specimens (4.5%) had been tested with lot #3, too few to permit any conclusions regarding possible variations in lot quality.

### No influence by HIV clade

Compared to HIV-1 subtype B, infection with CRF01_AE remained significantly associated with an increased proportion of false-recent results by Alg02, Alg03.2, Alg07 and, as a trend, Alg17. Closer inspection of the data showed that these associations were largely restricted to patients receiving HAART. For example, with Alg02, among treated patients, there were 17 false-recent results among 71 patients infected with CRF01_AE (24%) compared to 2 of 62 (3.2%) infected with subtype B (p = 0.0008, Fisher's exact test). In contrast, among untreated patients, the respective figures were 3/21 for CRF01_AE compared to 2/32 for subtype B (p = 0.37). Thus, only 3 of the 20 false-recent results were among treatment-naïve patients, too few to permit any safe conclusion.

Similarly, the apparent protective effects of infections by CRF02_AG or subtypes A, C and D also turned out to be associated with HAART. With Alg04, e.g., there were 13 false-recent results among 62 treated patients infected with subtype B (21%), while the respective numbers for CRF02_AG were 4/72 (5.6%). Thus, among treated patients, those infected with CRF02_AG had a significantly lower risk for false-recent results than those infected with subtype B (p = 0.009). In contrast, among untreated patients, the proportions of false-recent results between CRF02_AG (1/24, 4%) and subtype B (4/32, 12.5%) differed less (p = 0.38). Again, only 5 of the false-recent results occurred among HAART-naïve patients. Similar relationships were found with respect to the apparent protective effects of subtypes A, C and D compared to B (not shown).

**Figure 4:**
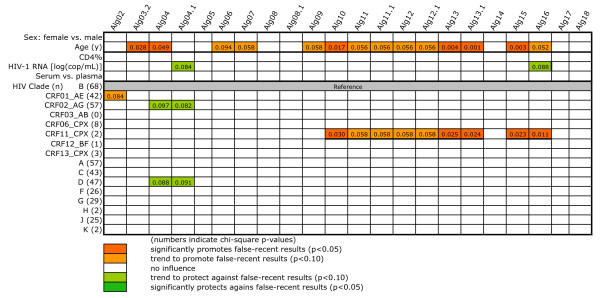
**Multivariate logistic regression analysis of factors that affect algorithm specificity among the 412 patients that are either HAART-naïve or exhibit HIV-1 RNA **≥**50 copies/mL despite HAART**. The meaning of the colors and numbers is as in the preceding figures. Odds ratios of variables of particular interest and their 95% confidence intervals are shown in the text. HIV-1 RNA was used as a continuous, logarithmized parameter, and concentrations below the lower limit of detection were set to 1 copy/mL.

In a next step to determine the relevance of the factors leading to false-recent results, we narrowed the analysis to those 412 patients who were either HAART-naïve or exhibited HIV-1 RNA ≥50 copies/mL despite receiving HAART (Figure 4). The analysis was further restricted to those independents which in Figure 3 had shown significant effects with at least two algorithms. Thus, CDC stage, HAART, sample volume, storage duration, modes of testing and scoring, and kit lot were no longer in the model. Alg03.1 had no false-recent result and could not be analyzed.

### Age impairs algorithm specificity

The only variable that retained broad significance in this setting was age, which significantly promoted false-recent results in 6 algorithms and showed a trend in a further 8. On average, the rate of false-recent results among these 14 algorithms increased by 5.2% for each additional year (range, 2.6 - 8.8%). CD4% lost all significance. HIV-1 RNA retained trends for significance with Alg 4.1 (OR, 0.74; 95% CI 0.52 - 1.04) and Alg16 (OR, 0.63; 95% CI 0.37 - 1.07). Although HIV-1 RNA was far away from significance in all other algorithms, the respective OR were usually below 1.0, particularly for all 16 combined algorithms, where the average OR per additional log RNA was 0.75. Thus, a certain influence of this parameter remains possible despite the lack of individual statistical significance. HIV clade also lost significance - note that the effect of CRF11_CPX with Algs 10 to 13.1, 15 and 16 is based on only 2 cases. Even more than in Figure 3, the remaining weak trends for either promoting or protective effects are based on too few cases to be of any relevance.

**Figure 5:**
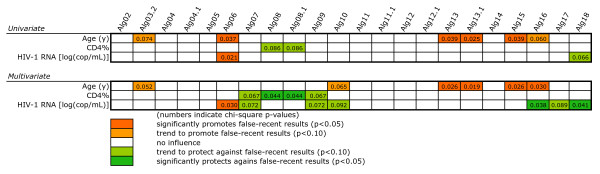
**Uni- and multivariate logistic regression analysis of factors that affect algorithm specificity among the 190 HAART-naïve patients**. The meaning of the colors and numbers is as in the preceding figures. Odds ratios of variables of particular interest and their 95% confidence intervals are shown in the text. HIV-1 RNA was used as a continuous, logarithmized parameter, and concentrations below the lower limit of detection were set to 1 copy/mL.

When finally focusing the investigation on the 190 HAART-naïve patients, univariate analysis revealed age as a factor, which significantly promoted false-recent results in four algorithms and showed a trend in two further ones (Figure 5, top panel). CD4% and HIV-1 RNA had no clear effects; a higher viral load even promoted false-recent results in Alg06. All other factors had no significance and are not represented in the figure. Multivariate analysis confirmed most effects of the three independents (lower panel). CD4% showed additional weak protective effects with Alg07 and Alg09, while HIV-1 RNA showed further protective effects with Algs 07, 09, 10, 16, and 17. Age lost its effect with Alg06, but gained a new one with Alg 10. Exclusion of the 6 cases with HIV-1 RNA <50 copies/mL led to the loss of all protective effects of HIV-1 RNA, while the effects of age and CD4% remained. This suggested that HIV-1 RNA <50 copies/mL promoted false-recent results also among untreated patients, while there was no effect among the higher concentrations. With regard to CD4%, close inspection of the data revealed no evidence for an association of low CD4% with low antibody intensities, and antibody intensities among patients in CDC stage C were on average higher than in stage A. Therefore, the weak effects of CD4% were not attributable to patients in advanced stage of disease.

In cross-comparison of Figures 3, 4 and 5, age clearly promoted false-recent results in all groups. Independent of the statistical significance in individual algorithms, the mean odds ratio for age among the algorithms differed little between the analyses of Figures 3, 4, and 5 and amounted to 1.021, 1.037 and respectively 1.032, thus suggesting a relative increase in false-recent results of about 3% per additional year of life. An HIV-1 RNA below 50 copies/mL promoted false-recent results in both treated and untreated patients; above this level there was, however, no effect of the concentration. With respect to CD4%, the strongly promoting effect in Figure 3 was strictly associated with long-term, successful HAART, as it was no longer present when HIV-1 RNA was above 50 copies/mL or when patients were HAART-naïve (Figure 5). If the weak protective effects in this latter group are real, they were not attributable to patients in the most advanced stage of disease. All other factors, including HIV-1 clade, had no effect.

## Discussion

The principal goal of the study was to determine the specificity of more than 20 Inno-Lia algorithms developed for estimating the fraction of recent infections in cohorts of HIV-1 infected patients [5]. A second aim was to identify possible factors that impair the specificity of the algorithms. Of particular importance was whether non-B clades of HIV-1 or advanced stages of immune deficiency would lead to false-recent results. These investigations are a first step of an ongoing overall evaluation of this new method, and without full knowledge of the sensitivity of the algorithms and their overall performance, which are investigated in a separate study to be published elsewhere, no definite conclusions should be drawn as to the suitability of this method for assessment of the recent infection rate in a population.

In order to answer the questions addressed in the present study, we retrospectively tested frozen specimens from well-characterized patients of the SHCS [18]. All patients, 86.8% of them selected for infection with non-B clades of HIV-1 and 73.4% receiving HAART for a median duration of more than one year, were in the chronic stage of infection and had been infected for longer than 12 months (Table 1). These 714 patients clearly represented older HIV infections as by our definition [5] and provided suitable conditions for an analysis of factors that affected algorithm specificity.

The high specificity of the 24 Inno-Lia algorithms (Table 2) already indicated that HIV-1 clade could have but a small effect. This was confirmed by univariate and multivariate logistic regression analysis (Figures 2 and 3). Both showed that the non-B clades that were available at sufficiently high numbers, i.e., subtypes A, C, D, F, G and J, as well as CRF01_AE, CRF02_AG and CRF06_CPX, did not affect the specificity in a relevant manner. Apparent promoting effects of CRF01_AE for false-recent results in Algs 02, 03.2, 07 and 17 were upon closer inspection found to be restricted to patients receiving HAART. Similarly, apparent protective effects of CRF02_AG and subtypes A, C and D were also restricted to patients receiving HAART. Both types of effects lost significance when the analysis was restricted to patients with no or only incompletely effective HAART. We conclude that these effects were largely treatment-associated and will not exert a sizeable effect in newly diagnosed, untreated patients.

In contrast to virus clade, there were some parameters, which affected the algorithms in a consistent and highly significant manner. One of these predictors was HIV-1 RNA <50 copies/mL, which was associated with a lower antibody intensity against all five HIV-1 antigens (Figure 1) and promoted false-recent results in most algorithms (Figure 3). HIV-1 RNA retained no significance when restricting the analysis to patients with no or incompletely effective HAART (Figure 4), thus confirming that only a very low or undetectable viral load would lead to false-recent results.

Our finding that HAART or, respectively, the low or undetectable viral load resulting from prolonged HAART was associated with a reduction in the concentration of HIV-specific antibodies in chronically infected patients is in contradiction to other reports. Although several studies have shown a delayed seroconversion, or partial seroreversion, in patients in whom HAART was started during acute infection or shortly thereafter [21-25], two studies of about 80 patients each found no reduction of HIV antibodies in chronically infected patients successfully treated with HAART for at least 5 years [24,25]. In contrast to these two studies and despite a shorter treatment duration, the average intensity of all five HIV antibody specificities in patients with <50 copies/mL HIV-1 RNA under HAART was significantly lower in the present study (Figure 1). This indicates a modest, but clear effect of HAART on antibody concentrations in chronically infected patients.

A high CD4% (or CD4+ count) was another factor strongly associated with false-recent results in the analysis of all 714 patients (Figure 3). This association is more difficult to understand and is probably the result of several superimposed effects. Further analysis revealed that patients who were receiving HAART and had higher CD4% than the median (≥21.4%) showed significant inverse correlations between CD4% and all antibodies except those to p24 (Spearman's rank correlation; p < 0.01 in all instances). In contrast, HAART-naïve patients or those with CD4% below the median did not exhibit such a correlation. In combination, these results may suggest that the association of high CD4% and low antibodies is an effect of HAART, whereby the patients that regain the highest CD4% are also those most likely to experience a decrease in their HIV-specific antibodies. The fact that CD4% had no significance in Figure 4 and even exhibited some protective effects in Figure 5 suggests that its promotion of false-recent results in Figure 3 is also a treatment-associated artifact. In HAART-naïve patients, a high CD4% may possibly protect against false-recent results in certain algorithms, but there was no indication that the patients with the most advanced disease were prone to false-recent results.

Sex, age, and testing serum instead of plasma were further frequent predictors of a false-recent result when investigated in the entire collective (Figure 3). Age promoted false-recent results in all algorithms that contained the term 'p31 = 0 AND p24≥2' (Algs 10 to 13.1, 15 and 16; see Table 2). Patients older than 35 years had twice as many false-recent results with Alg10 than the younger ones (9.9% vs. 4.6%, Fisher's exact test p < 0.01). They also exhibited a significantly lower mean intensity of p31 antibodies (p < 0.01). Age retained significance in the analyses of Figures 4 and 5 and exhibited similar average odds ratios between all analyzed groups. It is thus a factor that should also lead to some false-recent results in newly diagnosed patients. This finding fits into the well-known age-dependent weakening of the antibody responses to viral antigens such as present in viral vaccines [26-29].

Other factors including sex, using different lots of test kits, or testing serum instead of plasma, which appeared to affect the specificity of some algorithms when tested in all 714 patients (Figure 3), lost all significance when tested in HAART-naïve patients or those with a viral load ≥50 copies/mL despite HAART (Figure 4). As these factors are logically independent of HAART, they should also have no relevance when testing untreated patients.

### Limitations

For assessment of specificity of the algorithms and of factors that may affect specificity, a cohort of untreated patients would have been optimal. HAART has been the standard of care for patients with a certain degree of immunodeficiency for more than a decade, however, and it was impossible to meet this goal. Only 190 patients were HAART-naïve and only 20 of them were in CDC stage C.

Nevertheless, we consider our results to be valid for newly diagnosed, untreated patients, for the following reasons: Since HAART reduces the viral load and because an undetectable viral load in turn is associated with weaker antibodies, the antibody reactions in untreated patients will be stronger, which should result in even fewer false-recent results than found here. As a matter of fact, when the 714 patients were stratified according to CDC stage, the individual antibody intensities, as well as their sum, were higher in the untreated patients in all stages. Similarly, when stratification was for CD4% higher or lower than the median, the untreated patients had higher antibody intensities, except for p24, but the sum of all antibodies was higher again. This illustrates that single band patterns that would promote a false-recent result are successfully 'diluted out' or counteracted by suitably defined combination algorithms. Of note, some combination algorithms, in particular Alg14, but also Algs 11 to 13.1, appeared to be affected very little by all investigated variables.

Nevertheless, we cannot exclude the possibility that other factors than those investigated here may affect the specificity of the method. It is thus advisable to pre-determine the diagnostic performance of the test before transferring it to a new setting.

## Conclusions

The present study shows that the specificity of more than 20 Inno-Lia algorithms for recent infection is high. The specificity was clearly impaired by increasing age and an HIV-1 RNA load below 50 copies/mL, but not by the HIV-1 clade. Other variables, including sex, CDC stage, HAART without effective virus control, modalities of testing and result evaluation, did not matter. Similarly, for most algorithms there was no evidence for impairment by low CD4%. Some algorithms remained largely unaffected by all variables. We therefore expect that these algorithms should have a high specificity in all possible settings of untreated HIV-1 infected patients. Provided that they also exhibit a good diagnostic sensitivity and good overall performance, which are both assessed in a different study, they might become valuable tools for monitoring the rate of recent HIV-1 infections among newly diagnosed patients.

## List of abbreviations

STARHS: serologic testing algorithms for recent HIV seroconversion; Inno-Lia: the INNO-LIA^TM^ HIV I/II Score test; SHCS: Swiss HIV Cohort Study; HAART: highly active antiretroviral therapy; Alg, algorithm; OR: odds ratio;

## Competing interests

The authors declare that they have no competing interests.

Although the study was funded partially by Innogenetics NV, Ghent, Belgium, the manufacturer of the Inno-LiaTM HIV I/II Score test kit employed in the study, the decision to conduct the study was solely with the Swiss HIV Cohort Study (SHCS). The support of Innogenetics was restricted to conducting the testing of the study samples in the participating centers. All other costs were covered by the Swiss HIV Cohort Foundation and the Swiss Federal Office of Public Health, the public funder of the Swiss National Center for Retroviruses. None of the authors received - or will receive - any personal or institutional benefits of any kind.

The financial support by Innogenetics does in no way alter the authors' adherence to all policies of BMC on sharing data and materials. Thus, all data and knowledge conferred by the paper, once disclosed by their publication, will remain freely available to the public. All study data have already been transferred to the SHCS as the main public sponsor of the study.

## Authors' contributions

JS, LRB, SR, PB, MGo, IS, CA, SY, TK, and MR designed the study. CS, PB, SY and TK provided all HIV clade information. FSA, MR and JS selected the patients. LRB, SR, PB, MGo, IS, CA and GM conducted the testing and provided the Inno-Lia results. JS evaluated the data, produced tables and figures and wrote the first draft of the manuscript. MG, MR, FSA, MGo, LRB, SR, PB, IS, CA and MR critically reviewed and improved the manuscript. All authors read and approved the final manuscript.

## Pre-publication history

The pre-publication history for this paper can be accessed here:

http://www.biomedcentral.com/1471-2334/11/254/prepub

## References

[B1] Janssen RS Satten GA Stramer SL Rawal BD O'Brien TR Weiblen BJ New testing strategy to detect early HIV-1 infection for use in incidence estimates and for clinical and prevention purposes Jama 1998 280 42 48 10.1001/jama.280.1.42 9660362

[B2] Parekh BS McDougal JS Application of laboratory methods for estimation of HIV-1 incidence Indian J Med Res 2005 121 510 518 15817960

[B3] Murphy G Parry JV Assays for the detection of recent infections with human immunodeficiency virus type 1 Euro Surveill 2008 13 18775293

[B4] Le Vu S Pillonel J Semaille C Bernillon P Le Strat Y Meyer L Desenclos JC Principles and uses of HIV incidence estimation from recent infection testing--a review Euro Surveill 2008 13 18775292

[B5] Schupbach J Gebhardt MD Tomasik Z Niederhauser C Yerly S Burgisser P Assessment of recent HIV-1 infection by a line immunoassay for HIV-1/2 confirmation PLoS Med 2007 4 e343 10.1371/journal.pmed.0040343 18052604 PMC2100138

[B6] Parekh BS Kennedy MS Dobbs T Pau CP Byers R Green T Quantitative detection of increasing HIV type 1 antibodies after seroconversion: a simple assay for detecting recent HIV infection and estimating incidence AIDS Res Hum Retroviruses 2002 18 295 307 10.1089/088922202753472874 11860677

[B7] Tindall B Hing M Edwards P Barnes T Mackie A Cooper DA Severe clinical manifestations of primary HIV infection Aids 1989 3 747 749 10.1097/00002030-198911000-00010 2575913

[B8] Vento S Di Perri G Garofano T Concia E Bassetti D Pneumocystis carinii pneumonia during primary HIV-1 infection Lancet 1993 342 24 25 10.1016/0140-6736(93)91884-O 8100292

[B9] Tattevin P Camus C Arvieux C Ruffault A Michelet C Multiple organ failure during primary HIV infection Clin Infect Dis 2007 44 e28 29 10.1086/510683 17205433

[B10] Parekh BS Hu DJ Vanichseni S Satten GA Candal D Young NL Evaluation of a sensitive/less-sensitive testing algorithm using the 3A11-LS assay for detecting recent HIV seroconversion among individuals with HIV-1 subtype B or E infection in Thailand AIDS Res Hum Retroviruses 2001 17 453 458 10.1089/088922201750102562 11282014

[B11] Young CL Hu DJ Byers R Vanichseni S Young NL Nelson R Evaluation of a sensitive/less sensitive testing algorithm using the bioMerieux Vironostika-LS assay for detecting recent HIV-1 subtype B' or E infection in Thailand AIDS Res Hum Retroviruses 2003 19 481 486 10.1089/088922203766774522 12882657

[B12] Lange JM Goudsmit J De Wolf F Coutinho RA van der Noordaa J Serological markers in HIV infection Ann Med Interne (Paris) 1988 139 80 83 3293504

[B13] Schupbach J Haller O Vogt M Luthy R Joller H Oelz O Antibodies to HTLV-III in Swiss patients with AIDS and pre-AIDS and in groups at risk for AIDS N Engl J Med 1985 312 265 270 10.1056/NEJM198501313120502 2981407

[B14] Lange JM de Wolf F Krone WJ Danner SA Coutinho RA Goudsmit J Decline of antibody reactivity to outer viral core protein p17 is an earlier serological marker of disease progression in human immunodeficiency virus infection than anti-p24 decline Aids 1987 1 155 159 3126756

[B15] Pedersen C Nielsen CM Vestergaard BF Gerstoft J Krogsgaard K Nielsen JO Temporal relation of antigenaemia and loss of antibodies to core antigens to development of clinical disease in HIV infection Br Med J (Clin Res Ed) 1987 295 567 569 10.1136/bmj.295.6598.567 PMC1248741 3117234

[B16] Schmidt G Amiraian K Frey H Stevens RW Berns DS Densitometric analysis of Western blot (immunoblot) assays for human immunodeficiency virus antibodies and correlation with clinical status J Clin Microbiol 1987 25 1993 1998 2444624 10.1128/jcm.25.10.1993-1998.1987 PMC269384

[B17] Manca N di Marzo Veronese F Ho DD Gallo RC Sarngadharan MG Sequential changes in antibody levels to the env and gag antigens in human immunodeficiency virus infected subjects Eur J Epidemiol 1987 3 96 102 3497054 10.1007/BF00239745

[B18] Cohort Profile: The Swiss HIV Cohort Study Int J Epidemiol 2010 10.1093/ije/dyp321 19948780

[B19] Pollet DE Saman EL Peeters DC Warmenbol HM Heyndrickx LM Wouters CJ Confirmation and differentiation of antibodies to human immunodeficiency virus 1 and 2 with a strip-based assay including recombinant antigens and synthetic peptides Clin Chem 1991 37 1700 1707 1914169

[B20] Walther L Putkonen P Dias F Biberfeld G Thorstensson R Evaluation of HIV-1/HIV-2 immunoblots for detection of HIV-2 antibodies Clin Diagn Virol 1995 4 67 79 10.1016/0928-0197(94)00057-2 15566829

[B21] Jurriaans S Sankatsing SU Prins JM Schuitemaker H Lange J Van Der Kuyl AC Cornelissen M HIV-1 seroreversion in an HIV-1-seropositive patient treated during acute infection with highly active antiretroviral therapy and mycophenolate mofetil Aids 2004 18 1607 1608 10.1097/01.aids.0000131367.05823.ce 15238784

[B22] Kassutto S Johnston MN Rosenberg ES Incomplete HIV type 1 antibody evolution and seroreversion in acutely infected individuals treated with early antiretroviral therapy Clin Infect Dis 2005 40 868 873 10.1086/428127 15736021

[B23] Hare CB Pappalardo BL Busch MP Karlsson AC Phelps BH Alexander SS Seroreversion in subjects receiving antiretroviral therapy during acute/early HIV infection Clin Infect Dis 2006 42 700 708 10.1086/500215 16447118

[B24] Amor A Toro C Jimenez V Simon A Ramos B Soriano V Seroreversion of HIV antibodies in patients with prolonged suppression of viraemia under HAART AIDS 2006 20 1460 1462 10.1097/01.aids.0000233584.10209.43 16791025

[B25] Cornelissen M Jurriaans S Prins JM Bakker M van der Kuyl AC Absence of seroreversion in 80 HAART-treated HIV-1 seropositive patients with at least five-years undetectable plasma HIV-1 viral load AIDS Res Ther 2006 3 3 10.1186/1742-6405-3-3 16480525 PMC1395319

[B26] Powers DC Sears SD Murphy BR Thumar B Clements ML Systemic and local antibody responses in elderly subjects given live or inactivated influenza A virus vaccines J Clin Microbiol 1989 27 2666 2671 2592535 10.1128/jcm.27.12.2666-2671.1989 PMC267105

[B27] Stepanova L Naykhin A Kolmskog C Jonson G Barantceva I Bichurina M The humoral response to live and inactivated influenza vaccines administered alone and in combination to young adults and elderly J Clin Virol 2002 24 193 201 10.1016/S1386-6532(01)00246-3 11856620

[B28] Wolters B Junge U Dziuba S Roggendorf M Immunogenicity of combined hepatitis A and B vaccine in elderly persons Vaccine 2003 21 3623 3628 10.1016/S0264-410X(03)00399-2 12922091

[B29] Rendi-Wagner P Zent O Jilg W Plentz A Beran J Kollaritsch H Persistence of antibodies after vaccination against tick-borne encephalitis Int J Med Microbiol 2006 296 Suppl 40 202 207 16524776 10.1016/j.ijmm.2006.01.030

